# Graft-versus-host disease: establishing IL-33 as an important costimulatory molecule

**DOI:** 10.1172/JCI160692

**Published:** 2022-06-15

**Authors:** James Ferrara, Mariano Prado-Acosta

**Affiliations:** Tisch Cancer Institute, Icahn School of Medicine at Mount Sinai, New York, New York, USA.

## Abstract

Approximately half of patients with hematologic malignancy who are treated with allogeneic hematopoietic stem cell transplantation (alloHCT) experience graft-versus-host disease (GVHD), which has high mortality rates despite immunosuppressive therapy. IL-12 is known to drive donor T cells toward an inflammatory Th1 lineage in GVHD, but other mechanisms also promote pathological Th1 alloimmune responses. In this issue of the *JCI*, Dwyer et al. report on their use of transgenic mice and alloHCT models of GVHD to demonstrate that IL-33 acts directly on donor T cells to increase Tbet expression independently of IL-12. Notably, IL-33 amplified T cell receptor–signaling pathways and inhibited production of regulatory molecules. These findings firmly establish IL-33 as an important costimulatory molecule for Th1 cells during GVHD and provide a target for reducing GVHD, especially in the gastrointestinal (GI) tract, where damage drives mortality.

## T cells attack normal tissues

Allogeneic hematopoietic cell transplantation (alloHCT) is a life-saving therapy used to cure aggressive hematologic malignancies, such as acute leukemia and relapsed lymphoma. The curative potential of HCT derives from the elimination of cancer cells by the T lymphocytes in the donor graft, a process called the graft-versus-leukemia (GVL) effect. The antigenic stimuli for the donor T cells are histocompatibility antigens present on both normal and malignant cells of the host, and as a result, the donor T cells attack normal tissues as well. Thus, the beneficial GVL effect closely associates with graft-versus-host disease (GVHD), the major toxicity of alloHCT.

GVHD has both acute and chronic manifestations and is lethal when severe; approximately half of all alloHCT recipients experience either acute or chronic GVHD despite prophylaxis with combination immunosuppressive therapy. Acute GVHD affects three target organs: the gastrointestinal (GI) tract, the liver, and the skin (1). If GVHD fails to respond to immunosuppressive therapy with high-dose systemic corticosteroids, the prognosis is poor and mortality can exceed 50%. The GI tract is often the most resistant to treatment, and thus understanding the pathobiology of GVHD in the GI tract is an intensive focus of HCT research.

## The role of IL-33 in GVHD

Previous studies have shown an important role for IL–33 in the GI tract during the development of GVHD (2). Nonhematopoietic cells of the intestine are major sources of IL–33 in both clinical GVHD and experimental models, and the absence of IL-33 dramatically suppresses TNF-α in the serum and reduces GVHD severity in experimental models of alloHCT. Administration of IL-33 increases TNF-α production, increases the number of effector T cells in the lamina propria, and increases GVHD severity; administration of soluble ST2 (the IL-33 receptor) to block IL-33 binding to membrane-bound ST2 on donor T cells reduces inflammatory cytokines, attenuates GVHD damage to the GI tract, and improves overall survival. Yet IL-33 has a paradoxical role in the immunopathophysiology of various disease models where it can increase the stability and function of Tregs that act as an important break on inflammatory responses (3–5). Indeed, IL-33 increases the number of Tregs when administered to mice prior to alloHCT; these IL-33–expanded Tregs prevent the activation of macrophages and reduce the number of effector T cells that damage GVHD target tissues (6). Given these paradoxical effects of IL-33 on the severity of GVHD, it is critical to understand its molecular mechanism of action in greater detail.

**Figure 1:**
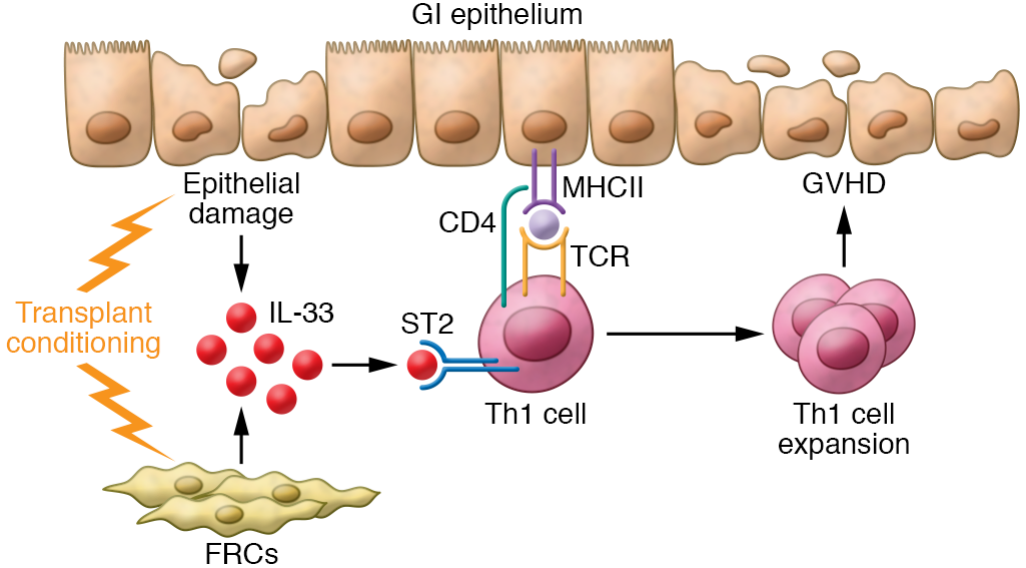
IL-33 promotes Th1 differentiation in GVHD. The alloHCT conditioning regimen damages the GI epithelium and FRCs that in turn produce IL-33, which binds to ST2 on donor Th1 cells. TCR recognition of allogeneic major histocompatibility complex (MHC) promotes Th1 cell differentiation and expansion, promoting GVHD and further epithelial damage.

IL-33 was thought to act primarily as an adjuvant to IL-12 produced by antigen presenting cells (APCs) that drive donor T cells toward an inflammatory Th1 lineage (7). In this issue of the *JCI*, Dwyer et al. demonstrate that IL-33 acts directly on T cells to increase Tbet expression independently of IL-12 (8). Their transplant conditioning regimen increased the expression of IL-33 in secondary lymphoid organs; IL-33 enhanced Th1 polarization and T cell expansion by amplifying T cell receptor–signaling pathways, particularly at low doses of alloantigen, and inhibiting production of regulatory molecules, such as IL-10 and FoxP3. Dwyer et al. (8) advance our understanding by firmly establishing ST2 as a costimulatory receptor for Th1 expansion that can increase GVHD severity after alloHCT (Figure 1).

## IL-33 and the GI tract in GVHD

The critical role of IL-33 in GVHD derives in part from its production in the intestine after alloHCT. IL-33 is released by both epithelial and mesenchymal cells, particularly fibroblastic reticular cells (FRCs) in secondary lymphoid organs of the intestine, known as the mesenteric lymph nodes. Seminal work by Koyama and colleagues has established a critical role for both nonprofessional APCs, particularly the mucosal epithelium, and professional APCs, such as dendritic cells, in the pathogenesis of acute GVHD (9, 10). The activation of APCs first in the epithelium and subsequently in mesenteric lymph nodes creates a cyclic inflammatory cascade, and this helps to explain why the GI tract is the GVHD target organ that is most difficult to treat and the greatest cause of mortality.

The importance of IL-33 in GVHD is also underscored by the finding that the serum concentration of soluble ST2 predicts GVHD severity and subsequent mortality. ST2 was first validated as a serum biomarker that predicted steroid-resistant acute GVHD (11). Subsequent work showed that combining ST2 analysis with a second serum biomarker, regenerating islet-derived 3-α, an antimicrobial peptide produced by Paneth cells, predicted long-term outcomes of GVHD (12–14). Each biomarker by itself has approximately the same predictive power, but only these two synergize effectively to predict long-term outcomes, probably because they reflect activation and damage of two separate components of the GI tract, the mesenchyme and epithelium (15). Thus, the combination of the two has been described as a liquid biopsy of the GI tract that can monitor intestinal GVHD damage and guide immunosuppressive therapy (16).

## Conclusions and future directions

The study by Dwyer et al. firmly establishes IL-33 as an important costimulatory molecule for Th1 cells in the GI tract during acute GVHD (8). Several questions deserve further consideration. Most importantly, how is IL-33, which is normally sequestered in the nucleus, released from cells and how can that process be inhibited to reduce GVHD? Second, what are the relative contributions of damaged GI epithelium and inflamed mesenchyme to the release of IL 33, and how do these processes relate to each other both temporally and spatially? Third, does IL-33 act as a similar costimulatory molecule for CD8^+^ T cells that also mediate acute GVHD? And does IL-33 play an important role in late acute GVHD by strengthening T cell receptor signaling when a small number of minor histocompatibility antigens in the host stimulate donor T cells several months following HCT? And finally, does manipulation of the IL-33/ST2 axis impair the GVL response, which determines the curative potential of alloHCT for hematologic malignancies? As future studies illuminate these issues, we may be able to devise therapeutic strategies to reduce or treat GVHD, especially in the GI tract, for which the unmet medical need is greatest and where progress is most likely to make alloHCT safer and more effective for all patients.

## Version 1 06/15/2022

Electronic publication
